# Effectiveness of Digital Interventions for Low-Income, Food-Insecure Populations: Natural Language Processing Study of WIC Smartphone App User Reviews, 2013-2024

**DOI:** 10.2196/78984

**Published:** 2025-12-31

**Authors:** Jihye Lee

**Affiliations:** 1 Stan Richards School of Advertising and Public Relations, Moody College of Communication, The University of Texas at Austin, 300 W Dean Keeton DMC 4.314 (A1200), Austin, TX, 78712, United States, 1 512 471 1101

**Keywords:** WIC, Special Supplemental Nutrition Program for Women, Infants, and Children, digital interventions, administrative burden, smartphone apps, COVID-19, natural language processing

## Abstract

**Background:**

The Special Supplemental Nutrition Program for Women, Infants, and Children (WIC) is a federal nutrition assistance program for low-income, food-insecure mothers and young children in the United States. Despite its intended goals, many eligible individuals forgo WIC benefits, in part due to administrative burden—defined as the complex, often frustrating processes encountered when navigating public benefit programs. In response, a range of digital interventions and policy waivers were introduced during the COVID-19 pandemic, but their effectiveness in reducing barriers remains unclear.

**Objective:**

Drawing from administrative burden theory and human-computer interaction research, this study examined user reviews of WIC smartphone apps (WIC Apps) used by local agencies. Specifically, it investigated (1) how obstacles to WIC access manifested in daily app use, (2) how user experiences shifted after the onset of the COVID-19 pandemic, and (3) how these changes were associated with app ratings.

**Methods:**

An original dataset of user reviews (N_review_=28,212) was compiled for 26 WIC Apps between 2013 and 2024. Structural topic modeling identified 8 key themes, and sentiment was examined with Robustly Optimized Bidirectional Encoder Representations From Transformers Pretraining Approach. Analyses compared topic prevalence and sentiment distributions before and after COVID-19. Mixed-effects models examined the relationship between topics, sentiment, and app ratings.

**Results:**

Technical concerns related to account authentication and login, document upload, and app updates were among the most prevalent themes. These issues were typically expressed with negative sentiment and appeared more frequently in pre–COVID-19 reviews than in post–COVID-19 reviews. Although reliability problems (eg, outages and maintenance) persisted, post–COVID-19 reviews increasingly emphasized features that facilitated program tracking, shopping and benefit redemption, and general ease of use, which were generally described with positive sentiment. Mixed-effects analyses indicated that these post–COVID-19 topics were significantly associated with higher app ratings (program tracking: B=0.21, SE=0.06; *P*=.001; shopping and redemption: B=0.18, SE=0.07; *P*=.01; and ease of use: B=0.10, SE=0.05; *P*=.04), whereas pre–COVID-19 concerns were not associated with ratings (*P*s>.05). When sentiment was added to the mixed-effect model, it became the dominant factor: negative sentiment was associated with lower ratings (B=−1.71, SE=0.03; *P*<.001), and positive sentiment was associated with higher ratings (B=1.78, SE=0.03; *P*<.001). After accounting for sentiment, no individual topic was significantly associated with ratings (*P*s>.05), suggesting that sentiment contributed to much of the variance previously linked to topics.

**Conclusions:**

User-centered digital interventions, such as WIC Apps, have the potential to support WIC access and participation.

## Introduction

### Administrative Burden in WIC

Food insecurity—defined as limited or uncertain access to nutritious food necessary for maintaining health—remains a pressing concern in the United States, affecting approximately 18 million households annually [1]. The Special Supplemental Nutrition Program for Women, Infants, and Children (WIC) addresses this challenge by providing supplemental food, nutrition education, and referrals to health care and social services for low-income, food-insecure individuals and families with young children (birth to age 5 years). Research shows that WIC contributes to alleviating health and social disparities. Participation in WIC has been associated with lower infant mortality rates, improved dietary quality, and enhanced cognitive development in children [2-4]. The program also yields significant economic benefits by reducing medical care expenditures for vulnerable families [5].

Despite its well-documented advantages, WIC faces a persistent take-up gap. According to the US Department of Agriculture [6], only 51.2% of eligible low-income households participate in WIC, and just 17.3% of enrollees fully redeem their benefits in a given month [7]. Research attributes much of this take-up gap to administrative burden, defined as the onerous processes individuals must navigate to access public benefits [8-11]. Administrative burden is typically conceptualized as 3 categories of costs. First, information costs refer to the time, effort, and resources required to learn about program availability, eligibility criteria, and application processes. Unlike universal benefits (eg, social security), need-based public benefit programs require proof of financial hardship, and complex documentation requirements or financial jargon often impose steep learning costs on applicants [12-14]. Second, compliance costs arise from fulfilling program requirements, such as submitting extensive documentation and attending in-person appointments for interviews, testing, and recertification [15]. Third, psychological costs involve stigma, anxiety, and loss of autonomy associated with public benefits. In the United States, discourses around poverty are shaped by neoliberal values that emphasize individual responsibility and justify burdensome procedures as safeguards against fraud [16,17]. Within this ideological framework, public benefit participants may internalize stigma and experience shame, anxiety, and moral scrutiny when accessing benefits.

In WIC, administrative burden manifests in multiple ways. For example, information and compliance costs are evident in the challenge of identifying WIC-approved food items. WIC provides monthly food packages that specify the types, brands, sizes, and quantities of food products eligible for purchase with WIC benefits. In practice, these items are often mislabeled or unavailable in stores, resulting in confusion, especially for participants with limited literacy or language skills [18]. Compliance costs also stem from mandatory nutritional education requirements. Typically provided through quarterly in-person counseling sessions, these classes offer guidance on healthy eating, breastfeeding support, and referrals to medical care. Although intended to promote long-term health, these requirements often burden participants with transportation barriers, inflexible work schedules, and limited caregiving support [19]. Psychological costs emerge in participants’ daily interactions with the program. Many participants report feelings of stigma and embarrassment at checkout, where benefits must be validated by store staff [20]. Taken together, information, compliance, and psychological costs of administrative burden create structural barriers that impede eligible participants from accessing WIC, navigating its requirements, and sustaining participation over time.

### Digital Interventions in Public Benefits: A Human-Computer Interaction Perspective

The growing recognition of administrative burden in accessing public benefits has spurred the adoption of digital interventions aimed at reducing barriers and improving benefit uptake. Many state WIC agencies, for instance, have implemented a variety of digital solutions, such as smartphone apps (hereafter referred to as WIC Apps) and online portals that allow participants to schedule appointments, submit documents, and track benefit balance remotely [21,22]. These initiatives parallel broader trends in digital service delivery, similar to telehealth services, in which technology is deployed to streamline bureaucratic processes and expand access.

Human-computer interaction (HCI) research offers a useful lens for examining how such digital systems can simultaneously reduce barriers and reproduce inequalities. HCI scholarship has long emphasized the importance of examining broader social and political systems, recognizing that technology is a sociotechnical product rather than a neutral tool [23-25]. This perspective has inspired critical examinations of how the design of digital systems may replicate and reinforce social inequalities, as well as explorations of how design interventions can improve users’ experiences. For example, participatory design [26], which emerged in the 1980s from Scandinavian workplace democracy movements, sought to redistribute design agency by involving end users as cocreators [27]. In parallel, Friedman’s framework of value-sensitive design [28,29] systematically incorporates values such as equity, dignity, and trust into sociotechnical systems. More recently, intersectionality [30] has guided scholars in investigating how overlapping systems of power, such as race, class, and gender, intersect to shape users’ interactions with technology. These approaches illustrate HCI’s longstanding engagement with justice in both design processes and outcomes.

Building on these theoretical foundations, a growing body of work investigates how digital systems mediate access to public resources for low-income and marginalized populations. Seminal work by Eubanks [31,32] shows that automated fraud detection and case management systems in public benefit programs embed administrative burden into routine bureaucratic interactions, functioning as a “digital poorhouse” that deepens inequality. Extending these critiques, recent studies demonstrate how technical design choices compound structural barriers to participation. For instance, Lee et al [33] demonstrated that public benefit websites and mobile apps are significantly less accessible than private financial service platforms. Barriers, such as complex authentication procedures, opaque error messages, and poorly localized interfaces, reveal how system design, rather than user literacy or technical skill, often determines whether low-income individuals can access essential benefits. Alongside these scholarly critiques, practice-oriented initiatives have sought to evaluate and improve digital service delivery. Code for America [34], for instance, has developed usability metrics to assess the inclusivity of public benefit programs and partnered with state governments to pilot interventions. One such initiative [35] used SMS text messaging to reduce information costs by providing direct links to resources and to lower psychological costs by framing benefit applications as entitlements rather than requests for help. In addition, this body of work highlights the dual nature of digital systems in public benefits: they can reproduce structural barriers and design harms; yet, they also offer opportunities for more human-centered, equitable interventions.

### This Study

This study investigates the effectiveness of digital interventions in the WIC program by analyzing user reviews of WIC Apps officially adopted by state and local agencies across the United States and affiliated territories. Prior research highlights both the promise of WIC Apps in reducing administrative burden and the persistence of barriers related to usability and infrastructure. Weber et al [36] provided one of the first systematic overviews (2017‐2018), identifying significant state-level variation in app deployment and noting that real-time shopping tools received the most favorable reviews. Administrative data from West Virginia later showed that WIC App use was associated with higher benefit redemption rates [37]. During the COVID-19 pandemic, policy waivers enabled remote access and prompted new research on program engagement. A multistate survey found that 79% of participants were satisfied with their state’s WIC Apps, though many suggested improvements such as benefit expiration reminders, chat functionality, and faster app performance [22]. Similarly, interviews in North Carolina highlighted the convenience of remote access but also persistent barriers like limited internet connectivity [38].

The expansion of digital interventions, coupled with COVID-19 policy waivers that enabled remote access to WIC, presents a novel context for examining participants’ experiences with barriers to access and how these experiences have changed over time [38]. The COVID-19 pandemic accelerated the adoption of digital solutions, as in-person program requirements became more difficult to meet. Federal support played a key role in facilitating this shift. In March 2020, the US Department of Agriculture and Congress introduced programmatic adjustments under the Coronavirus Aid, Relief, and Economic Security (CARES) Act, including physical presence waivers that suspended in-person requirements for enrollment, recertification, and quarterly nutrition counseling [39]. To further support modernization, the American Rescue Plan Act of 2021 allocated US $390 million to WIC’s digital infrastructure, followed by an additional US $100 million in 2023 [40,41].

Against this backdrop, this study analyzes a large, real-world dataset of user reviews (N=28,212) drawn from various WIC Apps deployed or used by state WIC agencies. Generated without researcher prompting, these reviews offer a rare opportunity to directly observe WIC participants’ digital experiences in natural contexts and at scale. Leveraging advances in natural language processing (NLP) for large-scale text analysis [42,43], this study uses structural topic modeling (STM) to identify themes in the user reviews and Bidirectional Encoder Representations From Transformers–based sentiment analysis to capture nuanced emotional valence. This integrated approach enables a scalable assessment of both content and sentiment in WIC participants’ experiences, highlighting NLP’s potential to inform understanding of digital health behaviors and equity in public service programs. The study addresses the following research questions (RQs):

- RQ1: How do WIC participants experience administrative burden while using WIC Apps?
- RQ2: How did user experiences with WIC Apps change between the pre- and post–COVID-19 periods?
- RQ3: How are WIC App user review topics and sentiment associated with overall app satisfaction?

## Methods

### Research Overview

**Figure 1.**
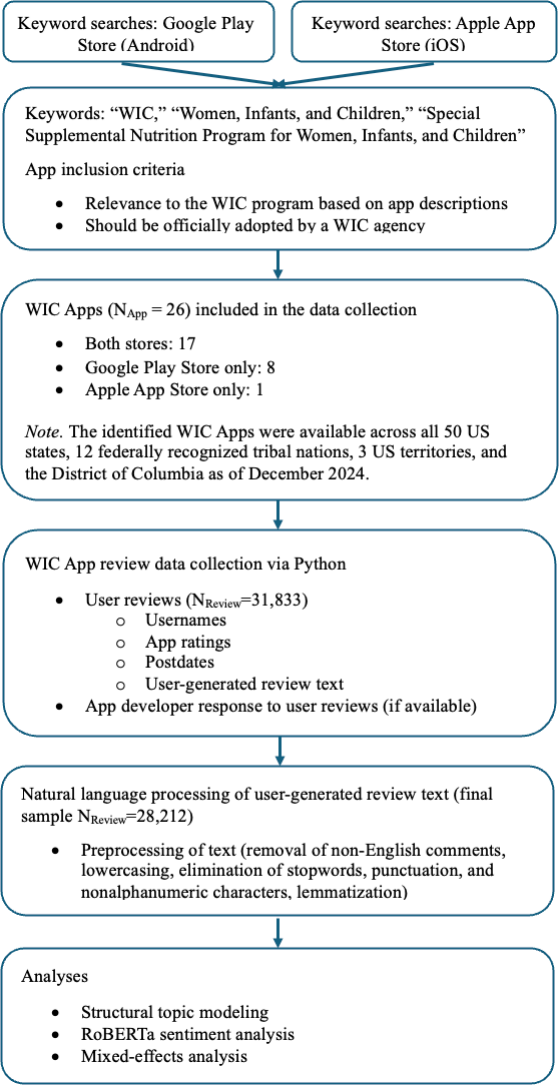
Research workflow. RoBERTa: Robustly Optimized Bidirectional Encoder Representations From Transformers Pretraining Approach; WIC: Special Supplemental Nutrition Program for Women, Infants, and Children; WIC App: WIC smartphone app.

As shown in Figure 1, the research began by compiling a list of WIC Apps available on the Google Play Store (Android) and Apple App Store (iOS). Since no consolidated list existed at the time of data collection (December 2024), 2 trained student researchers searched both app stores with 3 keywords: “WIC,” “Women, Infants, and Children,” and “Special Supplemental Nutrition Program for Women, Infants, and Children.” This list was further enriched by reviewing relevant literature and reports on WIC Apps [22,33,36].

App selection from this initial pool then followed 2 criteria: the app should be explicitly related to WIC and officially adopted by a WIC agency in the United States or its territories at the time of data collection (December 2024). To evaluate these criteria, app store descriptions were manually inspected to confirm relevance to WIC and evidence of agency adoption. Ambiguous cases were flagged and resolved in regular team meetings through systematic verification, which included conducting online searches for related news coverage, examining developer websites, and reviewing WIC agency websites. Duplicate listings by the same developer across app stores were merged to ensure that each app was represented only once in the analysis and avoid redundancy in the dataset. Apps that served broader benefit management functions (eg, combining WIC with SNAP, Medicaid, or other programs) were included as long as they were officially adopted by a WIC agency to ensure a comprehensive dataset reflecting the full range of digital tools used in WIC service delivery. Because app store review histories persist across version updates, the dataset also includes reviews referencing earlier iterations of the same app, provided the app remained active and available at the time of data collection. Reviews from fully deprecated, removed, or unofficial apps were excluded since their association with WIC agencies was either discontinued or could not be confirmed.

Following this structured selection and review process, a total of 26 WIC Apps were identified. These apps served all 50 states, 12 federally recognized tribal nations, the District of Columbia, and 3 US territories (American Samoa, Guam, and the Commonwealth of the Northern Mariana Islands).

Next, user reviews were collected from the identified WIC Apps using Python packages, such as Google Play Scraper and App Store Scraper. In compliance with platform policies, metadata was scraped, including anonymized usernames, app ratings, review dates, user-generated text comments (when available), and developer responses (when available). A total of 31,833 reviews were initially retrieved.

Standard NLP techniques were applied to prepare the data for analysis. Reviews were filtered to retain only English entries, converted to lowercase, and stripped of punctuation and emojis. Common stopwords (eg, “a,” “the,” and “and”), which add little semantic value, were removed. Lemmatization was then performed to reduce words to their base forms (eg, “children” → “child”). After cleaning, the final dataset included 28,212 user reviews: 25,961 from Android WIC Apps and 2251 from iOS WIC Apps. These reviews were posted between June 2013 and December 2024. The average app rating score was 3.11 (SD 1.75) on a 1‐5 scale.

The dataset included 26 WIC Apps, of which 17 were available on both Android and iOS, 8 were available only on Android, and 1 was available only on iOS. Several apps were adopted across multiple jurisdictions: for example, WICShopper served more than 30 states and tribal nations, while EzWIC supported participants in US territories and the Navajo Nation. Most apps (21/26, 80.8%) were developed and maintained directly by state health or human services agencies, while others were operated by nonprofit vendors (eg, myWIC Mosaic and MNC Go) or third-party service providers (eg, Bnft, WICShopper, and Providers). This mix highlights variation in governance and implementation, with some states relying on centralized government-developed platforms and others outsourcing to contracted vendors. Table S1 in Multimedia Appendix 1 presents detailed app-level data, including the WIC agencies using each app, platform availability, and the number of user reviews.

**Figure 2.**
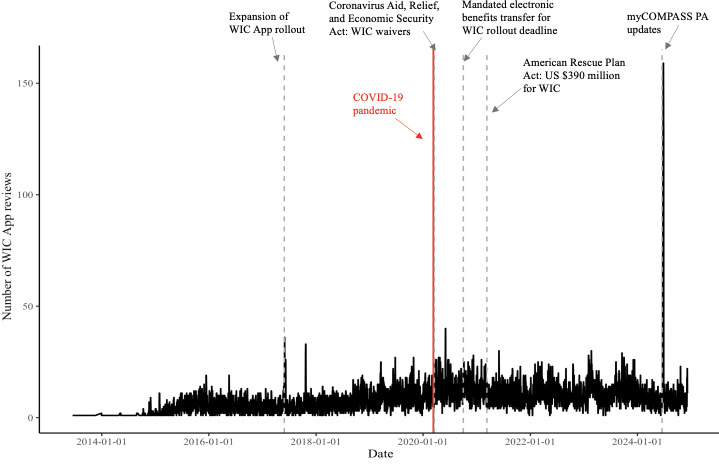
Longitudinal trend of WIC App user reviews and key milestones in WIC digital interventions. WIC: Special Supplemental Nutrition Program for Women, Infants, and Children; WIC App: WIC smartphone app.

Figure 2 illustrates the temporal distribution of reviews in the final dataset alongside major milestones in WIC digital transformation and COVID-19–related waivers. Review activity increased steadily after mid-2017 with the broader app rollout. A notable rise occurred after March 2020, reflecting expanded remote services under the CARES Act [44]. The most significant spike appeared in mid-2024 following technical disruptions to Pennsylvania’s myCOMPASS PA app. While COVID-19 marked a critical inflection point, the overall trajectory illustrates dynamic user engagement driven by both policy changes and technological developments.

### Structural Topic Modeling

#### Topic Modeling Approach

STM [45] was performed to identify key themes in user reviews. STM is a probabilistic NLP technique that uncovers latent topics in large text corpora by modeling the co-occurrence of words across documents (in this case, user reviews). Each document is represented as a mixture of topics, with estimated probabilities indicating the extent to which each topic is expressed in the text. Unlike traditional topic models, STM incorporates document-level metadata as covariates, enabling researchers to examine how topics differ across contexts such as time periods, populations, or settings. This feature makes STM particularly well-suited for this study, as it examines not only the themes that emerge in user reviews but also how topic prevalence differs between pre- and post–COVID-19 periods.

#### Model Selection

**Figure 3.**
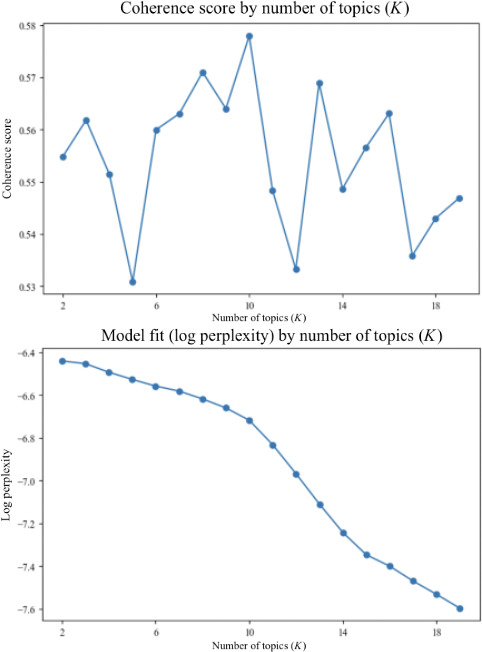
Model fit for topic models: semantic coherence (top) and log perplexity (bottom) by the number of topics.

To determine the appropriate number of topics (*K*) for STM, models were estimated with *K* ranging from 2 to 30, using 20 passes and 100 iterations each. Model fit was evaluated using semantic coherence and log perplexity scores (Figure 3). Topic-word lists and exemplar reviews were also inspected for interpretability. While larger *K* values improved perplexity, they reduced coherence and produced fragmented topics. Balancing these criteria, *K*=8 was selected, as it yielded coherent, distinct topics aligned with the study’s objectives.

After selecting *K*=8, model fit was assessed by comparing in-sample and held-out perplexity. The final 8-topic solution achieved a mean training perplexity of 123.94 (SD 0.47) and a held-out perplexity of 187.14 (SD 2.15). Although test perplexity was higher as expected [46], the relatively modest gap indicates that the model generalized reasonably well to unseen data. Topic stability was further examined by re-estimating the STM 100 times with different random seeds and aligning topics to the baseline model using cosine similarity of topic-word distributions. Topics demonstrated near-perfect reproducibility across runs (cosine similarity≈1.00), indicating a highly stable 8-topic solution [47].

#### Topic Label Generation

Topic labels were generated following a multistep process to ensure interpretability and reliability. First, preliminary topic names were developed based on the top 10 frequency-exclusivity (FREX) [48] terms and exemplar reviews. The FREX metric was selected, as it balances word frequency and distinctiveness across topics. Compared to alternatives, such as probability, which favors frequent words, or lift, which emphasizes rare words, FREX produces more interpretable topic labels by weighting both common and exclusive words.

Second, 2 experts in public health and digital interventions reviewed the proposed labels and suggested refinements. For example, expert feedback distinguished between program tracking (eg, checking benefit balance), document submission (eg, uploading documents required for recertification), and shopping and redemption features (eg, using a barcode scanner to check WIC-approved items), as these were likely to capture distinct types of administrative burden associated with accessing WIC, such as information, compliance, and psychological costs.

Third, using the refined set of 8 topic labels, a subset of reviews was coded by Prolific crowd workers, a platform widely used for academic research due to its high-quality responses [49]. To reduce cognitive burden, 3 balanced batches were created. Specifically, 5 reviews were randomly sampled from each topic to form a pool of 40 reviews per batch. To assess interbatch reliability, 10 reviews were duplicated across batches: 1 randomly selected from each of the 8 topics, with 2 topics randomly selected to contribute 2 reviews each. This resulted in 50 reviews with 40 unique reviews plus 10 overlapping reviews per batch. Each batch was coded independently by 2 workers. With 3 batches (6 workers in total), this design yielded 300 coded evaluations overall. Cohen κ within each batch indicated moderate to substantial agreement [50]: batch 1: κ=0.59; *z*=12.4; *P*<.001; batch 2: κ=0.61; *z*=10.7; *P*<.001; and batch 3: κ=0.67; *z*=14.7; *P*<.001. Fleiss κ across all 6 raters on the overlapping reviews indicated moderate agreement (κ=0.59; 95% CI 0.46-0.72; *P*<.001). Table 1 displays the refined topic labels with their top 10 FREX terms and exemplar reviews.

**Table 1.** Topic labels with top 10 frequency-exclusivity (FREX) terms and exemplar reviews.

Topic	Top 10 FREX terms	Exemplar reviews
Account authentication and login	account, password, time, number, new, let, log, cant, say, question	“It doesn’t even let you create an account,” “This phone number authentication is ridiculous. I don’t mind doing it for it to validate me for 48 hours or something, but literally every single time?”
Customer support	get, call, phone, dont, time, one, back, day, year, stamp	“I have called several times. I can’t get anyone on the phone. I waited on the phone hour and call hangs up.”
App reliability	work, great, always, doesnt, time, service, good, never, issue, well	“It’s never working, always a maintenance problem.”
App updates	update, cant, fix, even, say, wont, please, working, log, get	“After this new update i can not get into app.”
Shopping and redemption	balance, card, benefit, check, see, item, store, food, scan, love	“Very helpful when shopping in store. love that I can scan the barcode and see if it’s WIC approved.”
Program tracking	case, information, keep, need, help, know, benefit, track, also, great	“Very good way to keep track of your benefits as well as appointments with hra and keep you upto [cis] date with your case status.”
Ease of use	easy, love, use, much, thank, make, easier, convenient, great, helpful	“Simple, easy-to-use interface,” “App is user-friendly and quick.”
Document management	document, file, take, send, would, could, picture, better, fast, long	“Nice to be able to take photos and upload your documents instead of trying to fax them,” “When up loading [cis] files. Numerous photos has [cis] to be taken. Before a halfway decent can be submitted.”

### Sentiment Analysis

To examine how sentiment varied across topics, transformer-based sentiment analysis was applied to the reviews. Sentiment was classified as negative, neutral, or positive using the Cardiff NLP implementation of RoBERTa (Robustly Optimized Bidirectional Encoder Representations From Transformers Pretraining Approach) [51], a fine-tuned model on large-scale Twitter data to improve performance on short, informal texts such as app reviews. Both a categorical sentiment label and an associated probability score were computed for each review.

### Statistical Analysis

To address RQ1, each topic and its sentiment distribution were described with illustrative user reviews. Since STM provides the probability of each topic for individual reviews, the dominant topic of each review was identified as the one with the highest estimated probability. Using these assignments, overall topic prevalence was calculated as the proportion of reviews attributed to each STM topic, providing a descriptive baseline of the review corpus. Sentiment labels (negative, neutral, and positive), classified using RoBERTa, were then aggregated by dominant topic to calculate sentiment proportions within each topic.

In line with RQ2, two sets of analyses were conducted to examine changes in user experiences following the onset of COVID-19: (1) differences in topic prevalence and (2) differences in sentiment distributions within topics. Pre–COVID-19 reviews were defined as those submitted before March 11, 2020, the date the World Health Organization declared COVID-19 a global pandemic, and post–COVID-19 reviews were defined as those submitted afterward. For topic prevalence, the mean topic proportion was calculated for the pre- and postpandemic periods, and then the difference (post and pre) was computed along with a 95% CI using a normal approximation based on the pooled SE. Differences whose CIs excluded 0 were considered statistically significant. For sentiment distributions, chi-square tests of independence assessed whether the distribution of sentiment (negative, neutral, and positive) differed between pre- and post–COVID-19 reviews within each topic. Each review was assigned to the topic and sentiment category with the highest probability score. Cramér *V* was reported as an effect size.

For RQ3, mixed-effects models were used to examine the association between review topics, sentiment, and app ratings. This approach was selected to account for the nested structure of the data (individual reviews nested within apps and distributed across multiple review years). Since ratings may be correlated within the same app or time period, mixed-effects modeling helps address potential violations of the independence assumption underlying ordinary least squares regression [52]. Two mixed-effects models were specified. In the first model, topics identified through STM were included as independent variables predicting numeric app ratings. One topic—app updates—was excluded due to high multicollinearity (variance inflation factor [VIF]>10), which prevented model convergence and resulted in undefined coefficients. VIF diagnostics indicated that including this topic inflated VIF values beyond acceptable levels (threshold=10), whereas its removal reduced all VIFs to below 2, allowing stable estimation of the remaining 7 topics. After excluding the high-VIF topic, residual diagnostics (Figure S1 in Multimedia Appendix 1) showed no evidence of dispersion, though the Kolmogorov-Smirnov and outlier tests were significant, likely due to the large sample size. Visual inspection indicated mild deviations at the extremes, with no major systematic bias across predicted values.

In addition to STM topics, the second model included sentiment to capture both thematic and affective signals in the user reviews. Neutral sentiment was omitted to avoid perfect multicollinearity, since the 3 sentiment categories sum to 1. Accordingly, coefficients for negative and positive sentiment are interpreted relative to neutral sentiment.

Both mixed-effect models included state-level fixed effects to account for systematic differences in WIC implementation across states, such as local policies, infrastructure investment, and socioeconomic context. States were defined based on where an app was officially adopted by a WIC agency based on the app description. If an app was adopted by more than 1 state, it was coded as “Multiple States” (see Table S1 in Multimedia Appendix 1 for details on state categorization). Three control variables were also included to adjust for potential confounding factors in both models: presence of a developer response to reviews (present vs absent), total number of user reviews per app, and smartphone operating system (Android vs iOS). Finally, random intercepts were introduced for app and review year to account for clustering effects. This approach adjusts for unobserved heterogeneity by modeling within-app variation and between-app differences. Exploratory models tested random slopes for STM topics at both the app and year levels. While slopes for 2 topics (ease of use and shopping and redemption) modestly improved model fit according to the Akaike information criterion, they did not substantively alter the results and were penalized under the Akaike information criterion. For parsimony and model stability, the final models retained random intercepts for app and year only. Model estimation was conducted in R (R Foundation for Statistical Computing), using the *glmmTMB* package [53].

### Ethical Considerations

Prior to data collection, the study received approval from the institutional review board at The University of Texas at Austin (STUDY00003407). The approved protocol covered secondary analyses of publicly available, de-identified data without requiring additional consent. To protect participant privacy and confidentiality, no personally identifiable information was analyzed, and all data were aggregated for analysis.

## Results

### Topics and Sentiment in WIC App Reviews

To address RQ1, the STM analysis identified 8 distinct topics in user reviews of WIC Apps. Sentiment analysis further revealed how the tone of user experiences varied across these topics. Table 2 presents both the prevalence of each topic in the dataset and the distribution of sentiment (negative, neutral, and positive) within topics.

**Table 2.** Topic prevalence and sentiment distribution across WIC App^a^ reviews^b^.

Topic	Reviews, n (%)	Negative, n (%)	Neutral, n (%)	Positive, n (%)
App reliability	5379 (19.1)	4450 (82.7)	795 (14.8)	134 (2.5)
App updates	5134 (18.2)	2870 (55.9)	1408 (27.4)	856 (16.7)
Account authentication and login	3582 (12.7)	2371 (66.2)	1004 (28)	207 (5.8)
Document management	3133 (11.1)	1977 (63.1)	928 (29.6)	228 (7.3)
Customer support	2165 (7.7)	419 (19.4)	773 (35.7)	973 (44.9)
Shopping and redemption	3726 (13.2)	631 (16.9)	1116 (30)	1979 (53.1)
Ease of use	2841 (10.3)	720 (25.3)	1231 (43.3)	890 (31.3)
Program tracking	2252 (8)	441 (19.6)	912 (40.5)	899 (39.9)
Total number of observations	28,212 (100)	13,879 (49.2)	8167 (28.9)	6166 (21.9)

a WIC App: Special Supplemental Nutrition Program for Women, Infants, and Children smartphone app.

b Topic prevalence is based on the dominant topic per review. Sentiment distribution within each topic is derived from the Robustly Optimized Bidirectional Encoder Representations From Transformers Pretraining Approach classification.

App reliability was the most prevalent theme, representing nearly 1 in 5 (5379/28,212, 19.1%) reviews. This topic leaned strongly negative (negative: 4450/5379, 82.7%; neutral: 795/5379, 14.8%; positive: 134/5379, 2.5%), reflecting persistent frustrations with outages, downtimes, and maintenance problems. Users described these disruptions as major barriers to accessing benefits: “Never works! Always working on the website and I can never apply online. I have no transportation and I tried to call over the phone to apply and they said they do not do that anymore!,” “Always frozen, down for maintenance,” and “Trash. Never works properly so I miss deadlines or information because no one can send a letter anymore.” These reviews suggest that technical stability was a common concern for many WIC App users.

Issues related to app updates accounted for 18.2% (5134/28,212) of reviews, making this the second most frequently discussed theme. Sentiment was generally negative (negative: 2870/5134, 55.9%; neutral: 1408/5134, 27.4%; positive: 856/5134, 16.7%). Users reported unsuccessful troubleshooting or declines in app performance after updates. Illustrative comments included: “Worthless app since the last update” and “Terrible after the update! I’ve tried deleting and reinstalling twice, and nothing works.”

More than 1 in 10 (3582/28,212, 12.7%) reviews were related to account authentication and login, largely expressed with negative sentiment (negative: 2371/3582, 66.2%; neutral: 1004/3582, 28%; positive: 207/3582, 5.8%). Comments like “I hate having to reenter my information over and over again” and “Im [sic] registered but it keeps telling me invalid credentials. I went back over and over and retryed [sic] and I even changed my password more than once. But it still keeps saying the same thing and not letting me in. It is very irritating. 😡😠👿” indicate that repeated login failures, credential errors, and the time-consuming need to re-enter personal information were a primary barrier to accessing WIC.

Document management constituted 11.1% (3133/28,212) of reviews and was also a generally negatively evaluated topic (negative: 1977/3133; 63.1%, neutral: 928/3133, 29.6%; positive: 228/3133, 7.3%). Comments reflected both urgency and frustration related to the burden of uploading verification materials: “I’ve been trying to upload my documents for 4 days. Still not working! I was denied benefits last month because of this same issue!” and “I need to upload files now! Not everybody has access to a computer. There is a reason we are on benefits.” These frustrations illustrate the burden of compliance costs, meaning that participants had difficulty meeting program requirements due to technical challenges with document submission.

In contrast, customer support was the least prevalent topic, mentioned in 7.7% (2165/28,212) of reviews. Sentiment was largely positive or neutral (negative: 419/2165, 19.4%; neutral: 773/2165, 35.7%; positive: 973/2165, 44.9%). Some described dismissive or unhelpful service: “Zero customer service. You can’t call anyone to help you fix the problem” and “Call customer service, they’re rude as ever, makes me feel like I can’t get proper help without an attitude.” However, nearly half of the reviews in this category expressed satisfaction: “they will call you back if you leave a message very quickly” and “The ability to.use [sic] the app is wonderful. Customer service always on point.”

Shopping and redemption comprised 13.2% (3726/28,212) of reviews and was one of the most positively received topics (negative: 631/3726, 16.9%; neutral: 1116/3726, 30%; positive: 1979/3726, 53.1%). Users highlighted the convenience and efficiency of features that streamlined the shopping and redemption process, such as barcode scanners and digital benefit tracking. Comments like “If you are new to WIC, you wouldn’t understand how significant this app is! No more paper checks, no more arguing with the cashier” and “Finally a way to avoid being that awkward mama with the big WIC checks” illustrate how these features helped reduce administrative burden during shopping.

Ease of use accounted for 10.3% (2841/28,212) of reviews, with sentiment more balanced than other topics (negative: 720/2841, 25.3%; neutral: 1231/2841, 43.3%; positive: 890/2841, 31.3%). For example, users appreciated the app’s simplicity, user-friendly interface, and convenience: “It saves me a trip to the office, very convenient” and “Simple, easy-to-use interface.” Additionally, users valued the app’s ability to alleviate psychological costs stemming from the experience of welfare stigma: “Convenient, less embarrassing than standing in line with a bunch of small slips” and “So much better than dealing with rude women in the office!!” At the same time, some neutral and negative reviews pointed to areas where usability could be improved: “I like the idea off the app but sometimes doesn’t really work out the way u wanted 😉 but at least they provide us and easy way to going true the system.” and “It is useful, but the navigation and picture taking could be much improved.”

Program tracking was mentioned in 8% (2252/28,212) of reviews, representing the smallest proportion of topics. Sentiment was largely positive or neutral (negative: 441/2252, 19.6%; neutral: 912/2252, 40.5%; positive: 899/2252, 39.9%). Users praised its utility for checking benefit balances, tracking benefit payments, and monitoring appointments: “A super easy way to keep track of my benefits and what’s been used during the month,” “I’m always losing the receipt that shows what’s left, so it’s great to be able to check my phone,” and “It tells you when your appointments are and what you need to bring.”

As robustness checks, topic prevalence was compared across platforms (Android vs iOS; Table S3 in Multimedia Appendix 1), and a lexicon-based Valence Aware Dictionary and Sentiment Reasoner [54] sentiment analysis was compared to the RoBERTa models (Table S4 in Multimedia Appendix 1). To further assess sensitivity around a major policy milestone, topic prevalence was examined before and after the CARES Act was enacted (Table S5 in Multimedia Appendix 1). These analyses were largely consistent with the primary findings, supporting the robustness of the results.

### Shifts in WIC App Reviews: Pre- and Post–COVID-19

#### Changes in Topic Prevalence

**Figure 4.**
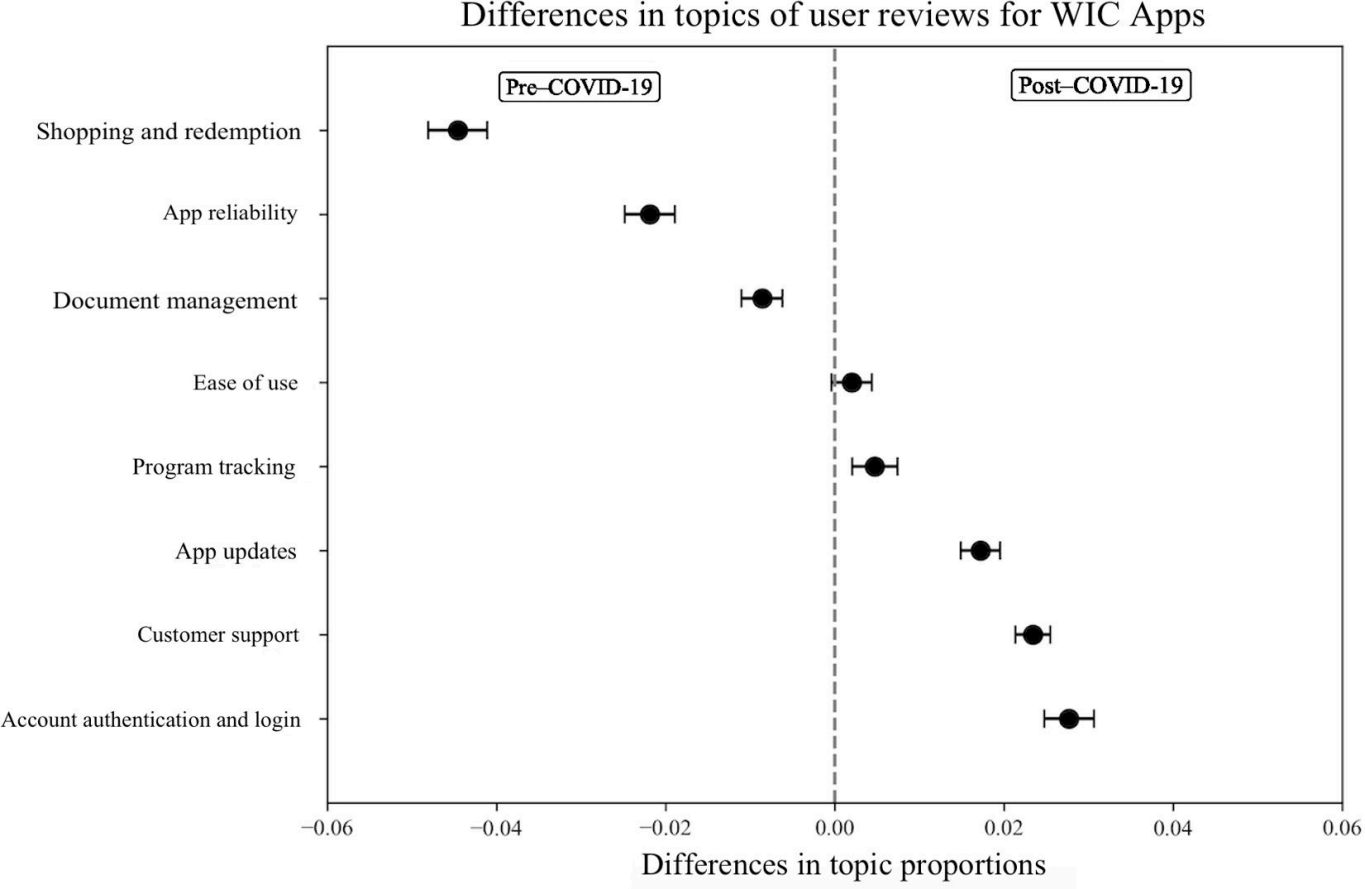
Topic differences in WIC Apps user reviews, pre- and post–COVID-19: a structural topic modeling approach. The x-axis represents the difference in topic proportions, with negative values indicating higher prevalence before COVID-19 and positive values indicating higher prevalence after COVID-19. The y-axis lists topics identified in user-generated reviews. Error bars represent 95% CIs. WIC: Special Supplemental Nutrition Program for Women, Infants, and Children; WIC App: WIC smartphone app.

To address RQ2, differences in topic prevalence were examined between reviews submitted before and after the onset of the COVID-19 pandemic (Figure 4). Topics that appeared more frequently in pre–COVID-19 reviews are displayed to the left of the dashed line, while those that became more common in post–COVID-19 reviews are shown to the right.

Several topics were significantly more common prior to COVID-19. Reviews that emphasized app updates and account authentication and login appeared more frequently in pre–COVID-19 reviews than post–COVID-19 reviews, highlighting the technical barriers that hindered benefit access during this period. Customer support and document management were also more prevalent before the COVID-19 pandemic, reflecting user experiences with compliance requirements (eg, uploading verification documents) and with seeking assistance from program staff.

App reliability showed little change between pre- and post–COVID-19. Reviews consistently reflected frequent outages, freezes, and downtimes. This stability suggests that while the pandemic altered the salience of some app features, underlying reliability problems remained a continuous barrier to access.

Other topics became more prominent after COVID-19. Program tracking increased in prevalence, as users relied on the app to monitor benefits and appointments. Shopping and redemption, particularly barcode scanners, also became more central after COVID-19, suggesting that users increasingly used these tools in the shopping experience. Likewise, ease of use was more frequently discussed in the post–COVID-19 period, reflecting appreciation for convenient, user-friendly design during a time when minimizing in-person contact was particularly important.

#### Sentiment Changes by Topic

Chi-square tests of independence were conducted to examine whether sentiment distributions differed between pre- and post–COVID-19 reviews. Full results are provided in Table S2 in Multimedia Appendix 1.

With the exception of app reliability, all topics showed significant changes in sentiment distributions between pre- and post–COVID-19, generally characterized by an increase in negative sentiment and a decline in positive sentiment, although effect sizes (Cramér *V*) were generally small to moderate. App reliability did not significantly differ across periods (*χ*^2^_2_=3.3; *P*=.19; *V*=0.02), with negative reviews remaining dominant (pre–COVID-19: 1983/2371, 83.6% vs post–COVID-19: 2467/3008, 82%). The largest shifts occurred for app updates (*χ*^2^_2_=67.4; *P*<.001; *V*=0.11), with negative sentiment rising from 48.1% (750/1559) to 59.3% (2120/3575) and positive sentiment decreasing from 22% (343/1559) to 14.3% (513/3575). Program tracking also exhibited notable changes (*χ*^2^_2_=57.9; *P*<.001; *V*=0.16), where negative reviews nearly doubled (133/1013, 13.1% to 308/1239, 24.9%) and positive reviews decreased (469/1013, 46.3% to 430/1239, 34.7%). Ease of use showed similar patterns (*χ*^2^_2_=51.8; *P*<.001; *V*=0.13), with negative reviews rising from 19.3% (249/1290) to 30.5% (471/1551) and positive reviews declining from 36% (465/1290) to 27.4% (425/1551). Customer support became more negatively evaluated as well (*χ*^2^_2_=20.5; *P*<.001; *V*=0.10), with negative reviews increasing (91/669, 13.6% to 328/1496, 21.9%) and positive reviews dropping (323/669, 48.3% to 650/1496, 43.4%).

Smaller but significant shifts appeared for account authentication and login (*χ*^2^_2_=19.2; *P*<.001; *V*=0.07) and document management (*χ*^2^_2_=9.1; *P*=.01; *V*=0.05), each reflecting modest increases in negative sentiment (912/1470, 62% to 1459/2112, 69.1% and 757/1263, 59.9% to 1220/1870, 65.2%, respectively) alongside decreases in positive evaluations. Shopping and redemption remained the most positively evaluated topic (*χ*^2^_2_=10.7; *P*=.005; *V*=0.05), with only minor changes (positive sentiment stable at approximately 53%).

### Impacts of User Review Topics on WIC App Ratings

To address RQ3, 2 mixed-effects models were estimated to examine how user review topics and sentiment were associated with app ratings. Table 3 presents the results of the model including only STM topics, whereas Table 4 reports the results of the full model that incorporated both STM topics and sentiment. Both models controlled for app-level characteristics, including states where each app was implemented, whether the developer responded to reviews, the total number of reviews for each app, and the app’s smartphone operating system (Android vs iOS).

**Table 3.** A mixed-effects model shows that topics reflecting positive user experiences are associated with higher app ratings^a^.

Fixed effects	App ratings	
	B (SE)	*P* value
Intercept	2.44 (0.38)	<.001
Topics of user reviews		
Account authentication and login	0.04 (0.06)	.56
Customer support	0.12 (0.07)	.09
Document management	0.07 (0.08)	.38
App reliability	−0.02 (0.07)	.83
Program tracking	0.21 (0.06)	.001
Shopping and redemption	0.18 (0.07)	.01
Ease of use	0.10 (0.05)	.04
State		
Alabama	0.08 (0.77)	.92
Arizona	−0.38 (0.46)	.41
Arkansas	0.55 (0.79)	.49
California	−0.89 (0.68)	.19
Delaware	−0.45 (0.80)	.57
Florida	0.19 (0.54)	.73
Georgia	0.30 (0.69)	.66
Indiana	−0.10 (0.69)	.88
Maryland	0.09 (0.70)	.90
Michigan	−0.58 (0.70)	.41
Minnesota	0.07 (0.58)	.90
New York	−0.35 (0.52)	.50
Oklahoma	0.20 (0.74)	.79
Pennsylvania	0.62 (0.68)	.36
South Carolina	−0.24 (0.70)	.73
South Dakota	−0.84 (0.75)	.27
Texas	0.06 (0.52)	.90
Wisconsin	0.59 (0.54)	.27
Developer response: present (reference=absent)	−0.83 (0.03)	<.001
Number of total reviews	<0.00001 (<0.00001)	<.001
Smartphone operating system: Android (reference=iOS)	0.01 (0.08)	.51

a Random effects: variance (WIC Apps)=0.37; variance (year of review postdate)=0.26. Model fit indices: Akaike information criterion=106,185.7; Bayesian information criterion=106,449.6; conditional *R*2=0.35; marginal *R*2=0.19. Number of observations=28,212. The analysis includes 28,212 user reviews nested within 26 WIC Apps, spanning 12 years (2013‐2024).

**Table 4.** A full mixed-effects model shows that sentiment of reviews is a robust predictor of app ratings^a^.

Fixed effects	App ratings	
	B (SE)	*P* value
Intercept	2.92 (0.17)	<.001
Topics of user reviews		
Account authentication and login	0.01 (0.04)	.84
Customer support	0.02 (0.05)	.73
Document management	−0.07 (0.05)	.23
App reliability	0.02 (0.05)	.71
Program tracking	0.07 (0.05)	.11
Shopping and redemption	0.06 (0.05)	.22
Ease of use	−0.03 (0.04)	.46
Sentiment of user reviews (reference=neutral)		
Negative	−1.71 (0.03)	<.001
Positive	1.78 (0.03)	<.001
State		
Alabama	−0.52 (0.38)	.92
Arizona	−0.17 (0.33)	.41
Arkansas	−0.03 (0.39)	.49
California	−0.49 (0.28)	.19
Delaware	−0.26 (0.40)	.57
Florida	−0.26 (0.23)	.73
Georgia	0.11 (0.28)	.66
Indiana	−0.19 (0.29)	.88
Maryland	−0.07 (0.29)	.90
Michigan	−0.34 (0.29)	.41
Minnesota	−0.07 (0.26)	.90
New York	−0.12 (0.22)	.50
Oklahoma	0.07 (0.34)	.79
Pennsylvania	0.33 (0.28)	.36
South Carolina	−0.24 (0.30)	.73
South Dakota	−0.68 (0.35)	.27
Texas	0.04 (0.22)	.90
Wisconsin	0.15 (0.23)	.27
Developer response: present (reference=absent)	−0.39 (0.02)	<.001
Number of total reviews	<0.00001 (<0.00001)	<.001
Smartphone operating system: Android (reference=iOS)	−0.12 (0.05)	.02

a Random effects: variance (WIC Apps)=0.06; variance (year of review postdate)=0.06. Model fit indices: Akaike information criterion=87,307.4; Bayesian information criterion=87,587.9; conditional *R*2=0.58; marginal *R*2=0.54. Number of observations=28,212. The analysis includes 28,212 user reviews nested within 26 WIC Apps, spanning 12 years (2013‐2024).

Table 3 indicates that topics that became more prominent in post–COVID-19 reviews were significantly associated with higher app ratings. Specifically, topics related to program tracking (B=0.21, SE=0.06; *P*=.001), shopping and redemption (B=0.18, SE=0.07; *P*=.01), and ease of use (B=0.10, SE=0.05; *P*=.04) were associated with higher ratings. In contrast, topics that were more common in pre–COVID-19 reviews were not significantly linked to ratings. Account authentication and login (B=0.04, SE=0.06; *P*=.56), customer support (B=0.12, SE=0.07; *P*=.09), document management (B=0.07, SE=0.08; *P*=.38), and app reliability (B=−0.02, SE=0.07; *P*=.83) all failed to reach statistical significance.

When sentiment was included in the model, its variance explained away the residual signal carried by topics, not because topics were unimportant, but because sentiment mediated their effects on ratings (Table 4). Specifically, negative sentiment was associated with significantly lower ratings (B=−1.71, SE=0.03; *P*<.001), whereas positive sentiment was linked to significantly higher ratings (B=1.78, SE=0.03; *P*<.001). In contrast, all individual review topics were not significantly associated with app ratings once sentiment was included in the model (*P*s>.05). These results suggest that while the specific program features and topics provided the content basis of reviews, the star ratings themselves were driven primarily by the emotional tone of reviews.

In both models, state-level indicators were not significantly associated with app ratings (*P*s>.05), suggesting that user experiences with WIC Apps were generally similar across states. One possible explanation is that many of the apps in the dataset were adopted by multiple state agencies, which may have produced relatively consistent user experiences across contexts. Notably, developer responses were associated with lower ratings across both models (without sentiment [Table 3]: B=−0.83, SE=0.03; *P*<.001; with sentiment [Table 4]: B=−0.39, SE=0.02; *P*<.001). This finding likely reflects the reactive nature of such responses, as developers typically reply to negative reviews rather than positive ones to engage dissatisfied users or provide remediation [55]. The total number of reviews per app was significantly associated with app ratings in both models, although the effect size was extremely small (Bs<0.00001, SEs<0.00001; *P*s<.001). No significant differences were observed between operating systems (Android vs iOS; *P*s>.05), indicating that satisfaction patterns did not systematically vary by platform.

To further ensure that the results are robust, the mixed-effects models were re-estimated, treating the ratings as an ordinal variable using a cumulative logit specification (Tables S6 and S7 in Multimedia Appendix 1). Results were largely consistent with the original Gaussian-based models, confirming the robustness of the main findings.

## Discussion

### Principal Findings

Leveraging the novel context that facilitated digital interventions and allowed for remote WIC services during the COVID-19 pandemic, this computational study investigated how WIC participants experienced administrative burden while using WIC Apps and how these experiences shifted with the expansion of remote access during COVID-19. Four main findings emerged.

First, consistent with administrative burden theory [8-10], a range of obstacles emerged in WIC App use. Particularly, technical barriers were a pervasive source of user frustration. Challenges with app reliability, account authentication, and login failures signaled that even initial access to WIC Apps was a significant hurdle. These problems did not end once participants gained entry: repeated difficulties with document management and update-related glitches compounded a burdensome experience throughout the user journey. Such obstacles produced not only logistical inconvenience but also psychological stress, sometimes diminishing users’ motivation to stay engaged: “I find it laughable that government agencies have the worst technology ... this was done by design.”

Second, despite persistent technical challenges, participants appreciated features that facilitated program access. Echoing prior research [22,36,56], barcode scanning was especially valued as a convenient, time-saving feature that helped reduce information and compliance costs (eg, identifying WIC-eligible food items) and mitigated psychological costs by lowering stigma during in-store transactions. Other widely appreciated features included real-time benefit tracking and appointment reminders, which aided users in managing schedules and avoiding benefit lapses. Importantly, these topics showed significant positive associations with app ratings, indicating that digital tools addressing information, compliance, and psychological burdens contributed to higher user satisfaction with service delivery. From an HCI perspective, these findings underscore how intentional design can create affordances that reduce administrative burden and improve usability for marginalized populations.

Third, user experiences shifted notably between the pre- and post–COVID-19 periods. Pre–COVID-19 reviews were dominated by technical concerns, such as login failures, document management, and technical glitches after updates. By contrast, post–COVID-19 reviews more often emphasized features tied to program tracking, shopping and redemption, and general ease of use. While these positive features appeared less frequently overall, they were framed positively and became more salient after the onset of COVID-19, when remote waivers and digital expansions occurred [38]. These shifts illustrate how policy and design changes can reshape the user experience of public service delivery.

Fourth, the mixed-effects analyses revealed that both topics and sentiment contributed to app evaluations, but their relative importance differed depending on model specification. When topics alone were included, features tied to program tracking, shopping, and redemption, and ease of use were significantly associated with higher app ratings. These results suggest that users valued design elements that were likely to reduce administrative burden and improve convenience in daily program interactions. However, when sentiment was added to the model alongside topics, sentiment became the dominant factor associated with ratings, and the effects of individual topics were no longer significant. As expected, negative sentiment was associated with lower ratings, while positive sentiment was linked to higher ratings. These patterns indicate that although certain features may contribute to positive experiences, it is the affective response—how participants felt navigating the app—that ultimately shaped their evaluations.

Overall, these results advance our understanding of health and social inequalities by showing how obstacles to public benefits are manifested in everyday interactions with WIC Apps. Drawing on administrative burden theory and HCI research, the study positions WIC Apps as critical sites where technology, inequality, and government intersect. Routine digital tasks, such as logging in, uploading documents, or coping with updates, emerged as points of friction. At the same time, features such as barcode scanning, benefit tracking, and appointment reminders were widely appreciated, illustrating how thoughtful design can reduce stigma, save time, and ease administrative burden. By integrating administrative burden theory with perspectives from HCI, this study underscores that the success of digital public service delivery depends not only on functional reliability but also on whether systems are designed to meet the needs of the populations most reliant on them.

The findings also generate important insights for program administrators, policymakers, and civic technology developers seeking to enhance the effectiveness of digital interventions in public health benefit programs like WIC. The study highlights persistent technical challenges that disrupted WIC access before and after COVID-19. WIC App users frequently discussed unstable app-agency connections, glitches, and poorly communicated maintenance. These issues point to the need for sustained investment in digital infrastructure beyond initial app development. Agencies should offer real-time technical support and clear, user-friendly support after app updates. At the same time, developers should adopt more inclusive and proactive design practices. Potential improvements include built-in text-to-speech for users with low literacy or visual impairments, in-app surveys triggered by key events (eg, failed login attempts), multilingual feedback channels to accommodate linguistically diverse populations, and offline functionality for participants with limited internet access. Additional features such as “save device” authentication options for shared caregiving situations could further support diverse caretakers who may share WIC benefits.

The implications of this study can extend beyond WIC. Many WIC participants are concurrently enrolled in other means-tested programs such as SNAP and Medicaid, and the barriers identified here are common across programs [33,34]. These overlapping challenges can accumulate into a compounding “time tax” that weighs most heavily on marginalized populations [57]. Recognizing administrative burden as a systemic issue across benefit programs is critical, as those with the least capacity to navigate inefficiencies often face them most acutely. Lessons from WIC Apps therefore highlight the broader potential of digital public infrastructure to improve access across multiple programs and underscore the need for sustained public-sector investment to scale innovations across domains. Recognizing the role of policy design in shaping administrative burden highlights the need for coordinated investment that addresses both technological and structural barriers to participation across other public benefit programs.

### Limitations

There are some limitations to consider when interpreting this study. First, it relied on publicly available app reviews, which lack demographic identifiers and contextual information about WIC App users, such as race or ethnicity, gender, geography, immigration status, digital literacy, and access to infrastructure. Prior research indicates that administrative burden is amplified when intersecting with marginalized identities. For example, the historically racialized and gendered stereotype of the “welfare queen” has stigmatized low-income Black mothers in accessing public benefits [58]. Non-English–speaking participants and individuals with immigration backgrounds often encounter language barriers and fears related to immigration status, which can deter participation in WIC programs [59]. Rural residents may encounter distinct obstacles, including limited access to WIC-authorized vendors and inadequate broadband infrastructure, which constrain both physical and digital program access [33]. Future research should examine how digital interventions, such as WIC Apps, shape the experiences of different subpopulations, the barriers these groups face, and how such interventions may either exacerbate or mitigate challenges associated with WIC participation.

The absence of demographic identifiers also made it impossible to assess the hardships faced by different types of caregivers. While WIC participation is often framed around mothers, fathers, grandparents, and other caregivers also play important roles. A few reviews pointed to these challenges directly, such as “This phone number authentication is ridiculous ... My WIC card is linked to my wife’s phone number, so if I’m shopping and need to get onto the app, I have to get the code from her. In this condition, the app is worthless.” Future research should recognize the diversity of caregiving arrangements to better capture the nuanced contexts of obstacles experienced by different caregivers.

In addition, the data did not allow for direct measurement of users’ readiness or attitudes toward using WIC Apps. User reviews capture the experiences of those who have already engaged with these tools, but they do not reflect the perspectives of individuals who may be unwilling or unable to adopt them. The technology acceptance model [60] suggests that perceived usefulness and perceived ease of use are important predictors of technology adoption. Therefore, future studies should incorporate survey or interview data to capture users’ readiness, attitudes, and perceptions of WIC Apps, including the perspectives of individuals who choose not to adopt WIC Apps. Combining user reviews with direct measures about user readiness and perceptions would provide a more comprehensive understanding of both adoption barriers and postadoption experiences.

The analysis included only English-language reviews and did not consider how non-English–speaking participants interact with WIC Apps. In the dataset, 371 (1.2%) reviews were written in other languages, with Spanish accounting for the majority (316 reviews). The decision to focus on English texts was due to methodological constraints, as incorporating multilingual reviews would have required additional tools and calibration beyond the scope of this study. However, WIC serves a racially and ethnically diverse population: approximately 43% of participants identify as Hispanic or Latino, and nearly 57% as White, with smaller shares represented by American Indian (5.8%), Asian (3.8%), Black (21.5%), Pacific Islander (0.8%), and multiracial (6.1%) participants [61]. Given this diversity, limiting the analysis to English-language reviews introduces bias and can obscure challenges faced by non-English users, such as navigating apps without multilingual support, fears linked to immigration enforcement, or cultural differences in dietary guidance and nutrition education [62,63]. To assess whether excluding non-English reviews might have affected substantive conclusions, a subset of non-English reviews (n=50, ≈13.5% of non-English reviews) was randomly selected and translated using Google Translate. Overall, similar themes emerged compared to the English-language analyses. For example, “Descargue la aplicación y nunca me dejó ingresar” (translated: “I downloaded the app and it never let me log in”) reflects account authentication and login, “De repente no sirve la aplicación” (translated: “Suddenly the app doesn’t work”) corresponds to app functionality, “Me ayuda bastante a la hora de buscar el producto que es elegible para WIC” (translated: “It helps me a lot when looking for WIC-eligible products”), aligns with shopping and redemption, and “Es práctico y fácil de usar” (translated: “It’s practical and easy to use”) relates to ease of use. Some reviews also highlighted challenges unique to non-English users, including linguistic barriers, as in “Tendría como cambiar de idioma y no está no sirve” (translated: “I would like to change the language, and this doesn’t work”), and potential racial discrimination, as in “no ayudan, discriminan solo por la raza” (translated: “They don’t help, they discriminate only by race”). Future studies should analyze multilingual reviews to better understand the experiences of linguistically and culturally diverse populations and to assess whether WIC Apps are equitably serving diverse communities.

While state-level fixed effects were included in the models, the study could not isolate how differences in specific COVID-19–related waiver dates for each state influenced user experiences. Because app reviews do not contain contextual details about local program administration, it was not possible to disentangle state-specific impacts. In addition, the absence of timestamps for developer responses prevented assessment of the temporal ordering between ratings and responses, which could offer insights into the role of developer engagement. Future research combining digital trace data with administrative records, additional metadata on developer response timing, or experimental designs could more directly investigate how state-level policies and developer engagement shape digital benefit delivery.

Finally, the sample reflects only those users who voluntarily posted reviews, a group likely to be more engaged and more motivated to report extreme experiences (very positive or very negative) [64]. While analyzing a large dataset provides a strong foundation, this self-selection limits the representativeness of the results and may underrepresent individuals who face barriers to digital engagement. For instance, some users noted challenges navigating the app for those less technology-savvy or those who lack a reliable internet connection: “Too difficult for the elderly” and “I wish you could use this app without phone service or wifi.” To mitigate this bias, future work should triangulate with complementary data sources, such as administrative data on benefit redemption, in-app use analytics, or interviews and surveys with participants [33,37]. Combining methods would provide a more comprehensive and balanced understanding of how digital interventions shape WIC participation.

### Comparison With Prior Work

This study makes 3 primary contributions. First, it provides systematic assessments of WIC Apps, with important implications for social and health disparities. Prior research has shown that hunger and food insecurity are central drivers of health disparities [65-67], and that burdensome interactions with public health benefits can deepen these inequalities by limiting access to essential resources [33,68-71]. While emerging evidence suggests that digital platforms may reproduce some of the challenges associated with claiming public benefits [33,72], important gaps remain in understanding how WIC Apps shape participants’ interactions with the program. Existing research has largely relied on small-scale pilot apps, often with narrow geographic scopes, rather than state-endorsed WIC Apps used in real-world settings. Moreover, evaluations of WIC’s digital tools typically emphasize broad program outcomes, such as website-smartphone app compatibility, enrollment rates, and benefit redemption [34,37,73]. Relatively little research has examined how obstacles to WIC access are experienced in participants’ day-to-day interactions with the program or how these experiences have evolved since COVID-19. This study addresses this gap by examining how structural barriers to WIC can manifest and evolve in everyday digital interactions with WIC Apps.

Second, this paper builds on existing studies of WIC digital interventions but alleviates several barriers that have limited empirical progress. It remains a challenging task to provide comprehensive, in-depth assessments of the effectiveness of digital interventions due to the limited availability of relevant information. Often, participants’ challenges go unreported [33], and even basic information about available WIC Apps is not readily accessible to researchers [36]. Here, the study draws on user reviews that provide rich contextual insights into daily interactions with the WIC program in their natural environments. By centering the daily experiences of individuals who are often marginalized in formal program evaluations [65,66], this research assesses the impact of current interventions and helps inform best practices that align more closely with the real-world needs and challenges faced by low-income, food-insecure individuals.

Third, the findings provide valuable lessons for public health program managers, policymakers, and civic technology practitioners aiming to enhance digital service delivery and reduce access barriers. As digital platforms play an increasingly central role in delivering public benefits, evaluating their effectiveness becomes essential [33]. The results show that the expansion of WIC Apps and program waivers coincided with improved ratings. It is important to note that digital platforms did not fully eliminate administrative burden or technical challenges, but improvements in review sentiment and satisfaction suggest the potential of user-centered digital design to enhance access to public benefits. This highlights the importance of continued support to build a user-oriented safety net. These findings are particularly timely, given renewed discussions on the future of public benefits and persistent health disparities [11,74].

Taken together, this study suggests key lessons for different stakeholders. For researchers, the study demonstrates how administrative burden is embedded in routine interactions with WIC Apps while also showing that some obstacles can be mitigated through digital interventions. These findings underscore both the persistence of administrative burden and the potential for HCI-informed design to alleviate such barriers in government services. For policymakers, evaluations of digital public service delivery should go beyond traditional metrics (eg, benefit redemption rates) and incorporate direct user feedback to identify challenges as well as features that improve program access. For developers, the results highlight the critical need for inclusive, user-centered design that reduces obstacles and accommodates the needs of diverse populations.

### Conclusions

In summary, the findings demonstrate that WIC Apps have played an important role in shaping how participants experience administrative burden when accessing benefits. The results highlight entrenched barriers in federal nutrition programs as well as the potential of digital interventions to help alleviate these challenges. Together, the findings offer important insights for the future design and delivery of public assistance services.

## Supplementary material

10.2196/78984 Multimedia Appendix 1 Supplementary tables and figures presenting the full list of WIC apps included in the study and robustness and validation analyses for the structural topic modeling of user reviews.

## Abbreviations

CARES: Coronavirus Aid, Relief, and Economic Security
FREX: frequency-exclusivity
HCI: human-computer interaction
NLP: natural language processing
RoBERTa: Robustly Optimized Bidirectional Encoder Representations From Transformers Pretraining Approach
RQ: research question
STM: structural topic modeling
VIF: variance inflation factor
WIC: Special Supplemental Nutrition Program for Women, Infants, and Children
WIC App: WIC smartphone app
