# Supplementary material for: Effectiveness of Digital Interventions for Low-Income, Food-Insecure Populations: Natural Language Processing Study of WIC Smartphone App User Reviews, 2013-2024
Source: J Med Internet Res. 2025 Dec 31;27:e78984. doi: 10.2196/78984 (PMC12755294; doi:10.2196/78984)

**Appendix**

Table S1. Full list of WIC Apps included in the dataset.

| App Names | States, Tribal Nations, and U.S. Territories | Number of Reviews  *n* (%) | Operating System | App Developer |
| --- | --- | --- | --- | --- |
| Bnft | North Carolina, Illinois | 807 (2.9%) | Android, iPhone | Nonprofit (operated by a third-party service for benefit management) |
| GA DCSS | Georgia | 1,151 (4.1%) | Android | Georgia Department of Community Health |
| WICShopper | Alaska, Chickasaw Nation (Summer Lunch Program only), Colorado, Connecticut, Florida, Hawaii, Idaho, Iowa, ITC Arizona, ITC Nevada, Kansas, Kentucky, Louisiana, Maine, Massachusetts, Missouri, Mississippi, Montana, Nebraska, Nevada, New Hampshire, New Jersey, North Dakota, Ohio, Oklahoma, Oregon, Passamaquoddy Tribe Pleasant Point, Pennsylvania, Rhode Island, Tennessee, Utah, Vermont, Washington, West Virginia, Wyoming | 2,899 (10.3%) | Android, iPhone | State government agency (varies by state) |
| Alabama WIC Program | Alabama | 20 (0.1%) | Android | Alabama Department of Public Health |
| Delaware WIC for Participants | Delaware | 16 (0.1%) | Android, iPhone | Delaware Health and Social Services |
| Florida Health WIC | Florida | 271 (1.0%) | Android, iPhone | Florida Department of Health |
| Maryland WIC | Maryland | 207 (0.7%) | Android, iPhone | Maryland Department of Health |
| WIC2Go | New York | 403 (1.4%) | Android, iPhone | New York State Department of Health |
| South Carolina WIC | South Carolina | 177 (0.6%) | Android, iPhone | South Carolina Department of Health and Environmental Control |
| South Dakota WIC | South Dakota | 28 (0.1%) | Android, iPhone | South Dakota Department of Health |
| myWIC Mosaic | Acoma-Canoncito-Laguna, Cherokee Nation, Louisiana, New Mexico, Pueblo of Isleta, San Felipe Pueblo, Santo Domingo (Kewa) Pueblo, Eight Northern Pueblos, Zuni Indian Reservation | 76 (0.3%) | Android | Nonprofit (may vary, often collaboration with state agencies) |
| my Arkansas WIC | Arkansas | 17 (0.1%) | Android | Arkansas Department of Health |
| my Oklahoma WIC | Oklahoma | 36 (0.1%) | Android | Oklahoma State Department of Health |
| EzWIC | American Samoa, Arizona, Commonwealth of the Northern Mariana Islands, District of Columbia, Guam, Navajo Nation | 109 (0.4%) | Android, iPhone | State government agency (varies by state) |
| California WIC App | California | 909 (3.2%) | Android, iPhone | California Department of Public Health |
| Indiana WIC | Indiana | 364 (1.3%) | Android, iPhone | Indiana State Department of Health |
| WIC Connect | Michigan | 289 (1.0%) | Android, iPhone | Michigan Department of Health and Human Services |
| NYC ACCESS HRA | New York City | 5,242 (18.6%) | Android | New York City Human Resources Administration |
| myCOMPASS PA | Pennsylvania | 3,770 (13.4%) | Android | Pennsylvania Department of Human Services |
| my TEXAS WIC | Texas | 256 (0.9%) | Android, iPhone | Texas Health and Human Services |
| MyACCESS Wisconsin | Wisconsin | 643 (2.3%) | Android | Wisconsin Department of Health Services |
| Wisconsin MyWIC | Wisconsin | 98 (0.3%) | Android, iPhone | Wisconsin Department of Health Services |
| My Minnesota WIC App | Minnesota | 98 (0.3%) | Android, iPhone | Minnesota Department of Health |
| Your Texas Benefits | Texas | 9,157 (32.5%) | Android, iPhone | Texas Health and Human Services |
| MNC Go | California | 15 (0.1%) | iPhone | Nonprofit (Mother’s Nutritional Center) |
| Providers: EBT, Mobile Banking^a^ | All fifty states | 1,154 (4.1%) | Android, iPhone | Nonprofit for electronic benefits transfer (EBT) services including WIC |
| Total number of observations | | 28,212 | | |

*Note.* ^a^The app is listed as Propel EBT & SNAP Benefits on the Google Play Store for Android smartphones, but it is branded as Providers. Apps published by the same developer in both the Google Play Store and Apple App Store were treated as duplicates and merged to consolidate their user reviews.

Table S2. Changes in sentiment distribution by topic, pre- and post-COVID-19.

| Topic | Sentiment | Pre-COVID  *n* (%) | Post-COVID  *n* (%) | *χ²* (df) | *P* value | Cramér’s V |
| --- | --- | --- | --- | --- | --- | --- |
| App Reliability  (*n* = 5,379) | Negative | 1,983 (83.6%) | 2,467 (82%) | 3.31 (2) | 0.19 | 0.02 |
|  | Neutral | 337 (14.2%) | 458 (15.2%) |  |  |  |
|  | Positive | 51 (2.2%) | 83 (2.8%) |  |  |  |
| App Updates  (*n* = 5,134) | Negative | 343 (48.1%) | 2,120 (59.3%) | 67.41 (2) | < .001 | 0.11 |
|  | Neutral | 466 (29.9%) | 942 (26.3%) |  |  |  |
|  | Positive | 750 (22%) | 513 (14.3%) |  |  |  |
| Account Authentication & Login (*n* = 3,582) | Negative | 912 (62%) | 1459 (69.1%) | 19.20 (2) | < .001 | 0.07 |
|  | Neutral | 463 (31.5%) | 541 (25.6%) |  |  |  |
|  | Positive | 95 (6.5%) | 112 (5.3%) |  |  |  |
| Document Management  (*n* = 3,133) | Negative | 757 (59.9%) | 1220 (65.2%) | 9.14 (2) | 0.01 | 0.05 |
|  | Neutral | 405 (32.1%) | 523 (28%) |  |  |  |
|  | Positive | 101 (8%) | 127 (6.8%) |  |  |  |
| Customer Support  (*n* = 2,165) | Negative | 91 (13.6%) | 328 (21.9%) | 20.52 (2) | < .001 | 0.10 |
|  | Neutral | 255 (38.1%) | 518 (34.6%) |  |  |  |
|  | Positive | 323 (48.3%) | 650 (43.4%) |  |  |  |
| Shopping & Redemption  (*n* = 3,726) | Negative | 182 (14.4%) | 449 (18.2%) | 10.73 (2) | 0.005 | 0.05 |
|  | Neutral | 408 (32.4%) | 708 (28.7%) |  |  |  |
|  | Positive | 671 (53.2%) | 1308 (53.1%) |  |  |  |
| Ease of Use  (*n* = 2,841) | Negative | 249 (19.3%) | 471 (30.5%) | 51.78 (2) | < .001 | 0.13 |
|  | Neutral | 576 (44.7%) | 655 (42.2%) |  |  |  |
|  | Positive | 465 (36%) | 425 (27.4%) |  |  |  |
| Program Tracking  (*n* = 2,252) | Negative | 133 (13.1%) | 308 (24.9%) | 57.92 (2) | < .001 | 0.16 |
|  | Neutral | 411 (40.6%) | 501 (40.4%) |  |  |  |
|  | Positive | 469 (46.3%) | 430 (34.7%) |  |  |  |

*Note.* Sentiment distributions were compared between pre-COVID and post-COVID reviews using chi-square tests of independence. *Cramér’s V* is reported as an effect size.

Table S3. Platform differences in topic prevalence (Android vs iOS)

| Label | Estimate (Android − iOS) | Std. Error | *P* value |
| --- | --- | --- | --- |
| App Reliability | 0.08 | 0.03 | 0.02 |
| App Updates | 0.24 | 0.04 | < 0.001 |
| Account Authentication & Login | 0.03 | 0.03 | 0.34 |
| Document Management | -0.08 | 0.03 | 0.01 |
| Customer Support | -0.08 | 0.03 | 0.02 |
| Shopping & Redemption | 0.31 | 0.03 | < 0.001 |
| Ease of Use | -0.17 | 0.04 | < 0.001 |
| Program Tracking | -0.02 | 0.03 | 0.53 |

*Note*. Topic prevalence differences between Android and iOS reviews were tested using beta regressions, controlling for total review count, developer response, and state. While some topics (e.g., *Ease of Use, Document Management*, and *Customer Support*) were slightly less prevalent in Android reviews, and others (e.g., *App Reliability, App Updates,* and *Shopping & Redemption*) were slightly more prevalent, all key topics were identified on both platforms. Topics on *Account Authentication & Login* and *Program Tracking* did not differ significantly. Overall, the same set of topics emerged across platforms, suggesting that the primary thematic patterns of user concerns and experiences are largely robust to platform differences.

Table S4. Sentiment distribution of each topic: A VADER (Valence Aware Dictionary and sEntiment Reasoner) approach

| Topic | Reviews  *n* (%) | Negative  *n* (%) | Neutral  *n* (%) | Positive  *n* (%) |
| --- | --- | --- | --- | --- |
| App Reliability | 5,379 (19.1%) | 5,052 (93.9%) | 216  (4.0%) | 111  (2.1%) |
| App Updates | 5,134 (18.2%) | 1,941 (37.8%) | 1,165 (22.7%) | 2,028 (39.5%) |
| Account Authentication & Login | 3,582 (12.7%) | 1,410 (39.4%) | 1,114 (31.1%) | 1,058 (29.5%) |
| Document Management | 3,133 (11.1%) | 1,570 (50.1%) | 673 (21.5%) | 890 (28.4%) |
| Customer Support | 2,165  (7.7%) | 308  (14.2%) | 309 (14.3%) | 1,548 (71.5%) |
| Shopping & Redemption | 3,726 (13.2%) | 505  (13.5%) | 357  (9.6%) | 2,864 (76.9%) |
| Ease of Use | 2,841 (10.3%) | 715  (25.2%) | 516 (18.2%) | 1,610 (56.7%) |
| Program Tracking | 2,252  (8.0%) | 368  (16.3%) | 333 (14.8%) | 1,551 (68.9%) |
| Total | 28,212 (100%) | 11,869 (42.1%) | 4,683 (16.6%) | 11,660 (41.3%) |

Note. The overall pattern of sentiment in the VADER analysis mirrors the RoBERTa results. Technical topics such as *App Reliability, Account Authentication & Login*, and *Document Management* are predominantly negative, whereas topics related to user experience, including *Customer Support, Shopping & Redemption, Ease of Use*, and *Program Tracking*, show higher proportions of neutral or positive sentiment. VADER tends to classify some reviews more positively than RoBERTa, likely because lexicon-based methods are less sensitive to context and nuanced expressions of dissatisfaction. Despite these differences in magnitude, the overall patterns across topics remain consistent, supporting the robustness of the primary findings.

Table S5. Topic prevalence before and after the CARES Act in WIC App reviews

| Label | Est. | Std. Error | P- value | Interpretation |
| --- | --- | --- | --- | --- |
| App Reliability | -0.0004 | - | - | Model could not estimate change reliably due to insufficient variability |
| App Updates | -0.007 | 0.009 | 0.46 | No significant change |
| Account Authentication & Login | -0.003 | 0.007 | 0.62 | No significant change |
| Document Management | -0.005 | 0.005 | 0.32 | No significant change |
| Customer Support | -0.005 | 0.004 | 0.26 | No significant change |
| Shopping & Redemption | -0.002 | 0.006 | 0.74 | No significant change |
| Ease of Use | 0.017 | 0.009 | 0.06 | Marginal increase |
| Program Tracking | 0.009 | 0.004 | 0.03 | Small but statistically significant increase |

*Note*. A sensitivity check was conducted to assess differences in topic prevalence before and after the Coronavirus Aid, Relief, and Economic Security (CARES) Act, enacted on March 27, 2020. Est. = Estimate; Std. Error = Standard Error.

Table S6a. Mixed-effects cumulative logit model predicting app ratings from review topics

|  | App Ratings | | |
| --- | --- | --- | --- |
|  | B(Est.)^a^ | SE^b^ | *P* value |
| **Fixed effects** |  |  |  |
| Topics of user reviews |  |  |  |
| Account Authentication & Login | 0.04 | 0.51 | 0.61 |
| Customer Support | 0.16 | 1.95 | 0.051 |
| Document Management | 0.10 | 1.11 | 0.27 |
| App Reliability | 0.01 | 0.10 | 0.92 |
| Program Tracking | 0.25 | 3.30 | .001 |
| Shopping & Redemption | 0.17 | 2.05 | 0.04 |
| Ease of Use | 0.14 | 2.32 | 0.02 |
| State |  |  |  |
| Alabama | -0.97 | 0.82 | 0.24 |
| Arizona | -1.02 | 0.57 | 0.07 |
| Arkansas | -0.41 | 0.82 | 0.61 |
| California | -1.92 | 0.70 | 0.01 |
| Delaware | -2.13 | 0.95 | 0.02 |
| Florida | -0.65 | 0.56 | 0.24 |
| Georgia | -0.74 | 0.71 | 0.30 |
| Indiana | -1.16 | 0.71 | 0.10 |
| Maryland | -0.97 | 0.71 | 0.17 |
| Michigan | -1.64 | 0.71 | 0.02 |
| Minnesota | -1.24 | 0.59 | 0.04 |
| New York | -0.94 | 0.54 | 0.08 |
| Oklahoma | -0.75 | 0.77 | 0.33 |
| Pennsylvania | -0.12 | 0.71 | 0.87 |
| South Carolina | -1.34 | 0.71 | 0.06 |
| South Dacota | -2.26 | 0.81 | 0.01 |
| Texas | -0.51 | 0.54 | 0.34 |
| Wisconsin | -0.34 | 0.55 | 0.54 |
| Developer response: Present (Ref^c^. Absent) | -0.93 | -0.93 | < .001 |
| Smartphone operating system: Android (Ref^c^. iOS) | -0.42 | -0.42 | < .001 |
| **Random effects** |  |  |  |
| Variance (WIC Apps) | 0.40 | | |
| Variance (Year of the review postdate) | 0.32 | | |
| **Fit indices** |  | | |
| AIC^d^/ BIC^e^ | 73,132.89/73,405.06 | | |
| Log likelihood | -36,533.4 | | |

*Note*. As a robustness check, a cumulative logit mixed-effects model was estimated to investigate the relationship between app ratings and review topics. Since ordinal models are sensitive to model complexity and sparse data, the total number of reviews per app, one of the control variables in the original Gaussian model, was excluded to achieve stable convergence. Overall, estimated effects of topics were consistent with the Gaussian model reported in Table 3, showing negligible differences. ^a^B = estimates; ^b^SE = standard error; ^c^Ref. = reference category; ^d^AIC = Akaike information criterion; ^e^BIC = Bayesian information criterion; Two control variables were included: presence of developer responses and smartphone operating system (Android vs. iOS).

Table S6b. Mixed-effects cumulative logit model predicting app ratings from review topics and sentiment scores

|  | App Ratings | | |
| --- | --- | --- | --- |
|  | B(Est.)^a^ | SE^b^ | *P* value |
| **Fixed effects** |  |  |  |
| Topics of user reviews |  |  |  |
| Account Authentication & Login | 0.001 | 0.02 | 0.99 |
| Customer Support | 0.01 | 0.11 | 0.91 |
| Document Management | -0.14 | -1.35 | 0.18 |
| App Reliability | 0.05 | 0.49 | 0.62 |
| Program Tracking | 0.13 | 1.55 | 0.12 |
| Shopping & Redemption | 0.05 | 0.59 | 0.55 |
| Ease of use | -0.04 | -0.55 | 0.58 |
| Sentiment of user reviews (Ref^c^. Neutral) |  |  |  |
| Negative | -2.40 | -41.26 | < .001 |
| Positive | 2.81 | 49.61 | < .001 |
| State |  |  |  |
| Alabama | -1.52 | -2.25 | 0.02 |
| Arizona | -0.65 | -1.03 | 0.30 |
| Arkansas | -0.50 | -0.74 | 0.46 |
| California | -1.24 | -2.45 | 0.01 |
| Delaware | -1.36 | -1.66 | 0.10 |
| Florida | -0.75 | -1.82 | 0.07 |
| Georgia | -0.35 | -0.70 | 0.48 |
| Indiana | -0.81 | -1.57 | 0.12 |
| Maryland | -0.54 | -1.02 | 0.31 |
| Michigan | -1.04 | -2.00 | 0.05 |
| Minnesota | -0.72 | -1.57 | 0.12 |
| New York | -0.50 | -1.28 | 0.20 |
| Oklahoma | -0.23 | -0.38 | 0.71 |
| Pennsylvania | 0.14 | 0.28 | 0.78 |
| South Carolina | -0.79 | -1.50 | 0.13 |
| South Dacota | -1.89 | -2.82 | 0.00 |
| Texas | -0.24 | -0.62 | 0.54 |
| Wisconsin | -0.15 | -0.37 | 0.71 |
| Developer response: Present (Ref^c^. Absent) | -0.61 | -15.75 | < .001 |
| Smartphone operating system: Android (Ref^c^. iOS) | -0.49 | -5.78 | < .001 |
| **Random effects** |  |  |  |
| Variance (WIC Apps) | 0.2 | | |
| Variance (Year of the review postdate) | 0.17 | | |
| **Fit indices** |  | | |
| AIC^d^/ BIC^e^ | 57,788.84/ 58,077.5 | | |
| Log likelihood | -28,859.42 | | |

*Note*. As a robustness check, a cumulative logit mixed-effects model was estimated to examine the relationship between app ratings, review topics, and sentiment scores. Due to the sensitivity of ordinal models to model complexity and sparse data, the total number of reviews per app (a control variable included in the original Gaussian model) was excluded to ensure model convergence. The effects of topics and sentiment scores remained consistent with the Gaussian model results (see Table 4). ^a^B = estimates; ^b^SE = standard error; ^c^Ref. = reference category; ^d^AIC = Akaike information criterion; ^e^BIC = Bayesian information criterion; Two control variables were included: presence of developer responses and smartphone operating system (Android vs. iOS).

Figure S1. Quantile-Quantile plot of model residuals.


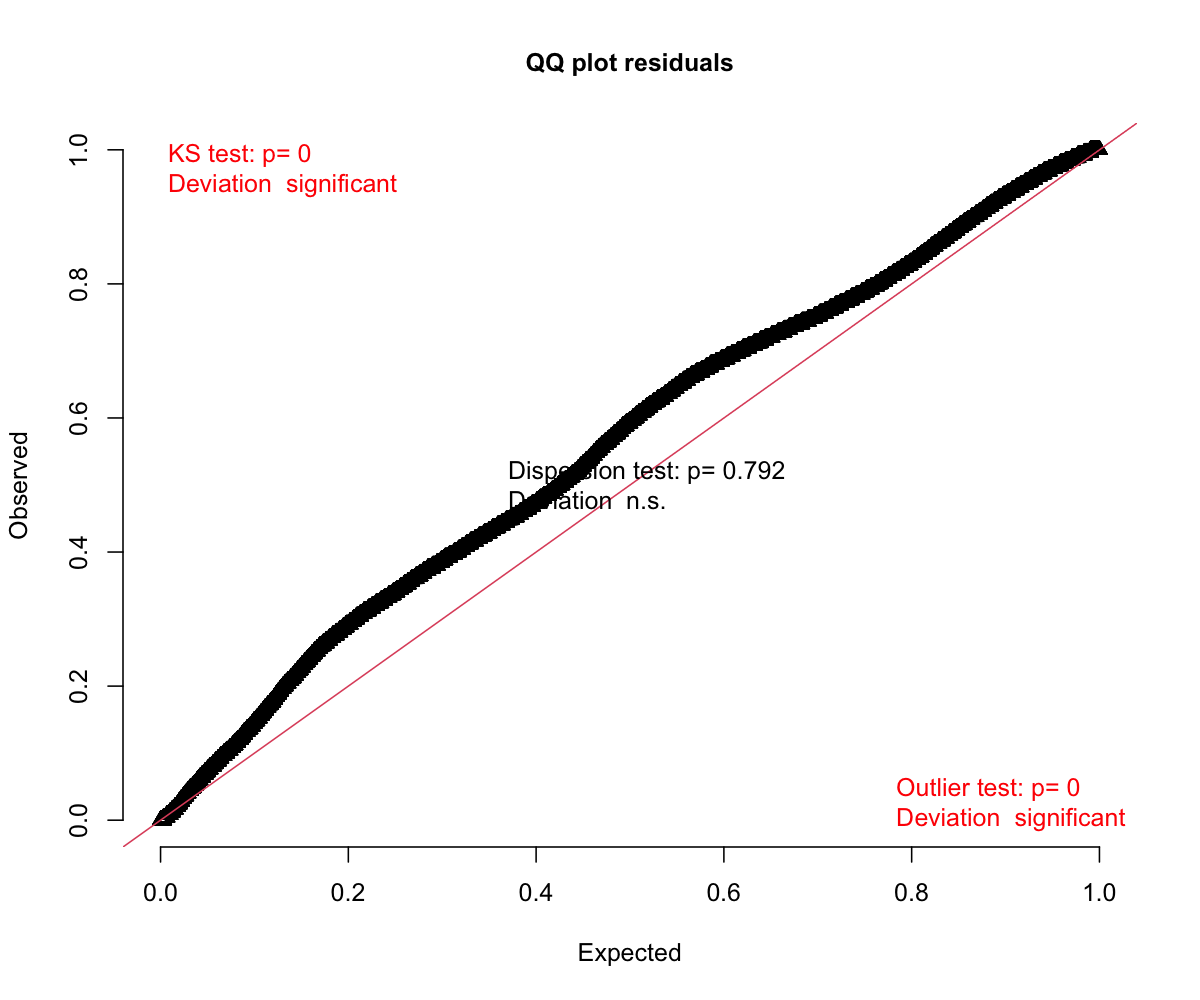

Supplement: Multimedia Appendix 1 [file jmir-v27-e78984-s001.docx]
